# *Oligella* spp.: A systematic review on an uncommon urinary pathogen

**DOI:** 10.1007/s10096-024-04797-9

**Published:** 2024-04-26

**Authors:** Eric Farfour, Marc Vasse, Alexandre Vallée

**Affiliations:** 1 Service de biologie Clinique, Hôpital Foch, 40 rue Worth, Suresnes, 92150 France; 2 Université Paris-Saclay, INSERM Hémostase inflammation thrombose HITH U1176, Le Kremlin-Bicêtre, 94276 France; 3 Département d’Epidémiologie et de Santé Publique, Hôpital Foch, Suresnes, France

**Keywords:** *Oligella urethralis*, *Moraxella urethralis*, *Oligella ureolytica*, CDC group IVe, Urinary tract infection

## Abstract

**Background:**

*Oligella* is an uncommon Gram-negative coccobacillus that was first thought to belong to the urogenital tract. The genus *Oligella* comprises two species that were recovered from various samples worldwide.

**Methods:**

We perform a systematic review focusing on *Oligella* microbiological characteristics, habitat, role in Human microbiome and infection, and antimicrobial susceptibility.

**Results:**

In humans, *Oligella* is mainly found as part of the microbiome of individuals with predisposing conditions. *Oligella* were also associated with invasive infections in patients with underlying diseases. Nevertheless, their prevalence remains to determine. *Oligella* culture requires up to 48 h on agar media in vitro, while urinary samples are usually incubated for 24 h. Consequently, microbiologists should be prompt to prolong the incubation of agar media when the direct examination showed Gram-negative coccobacilli. *Oligella* is accurately identified using MALDI-TOF mass spectrometry, but biochemical methods often provided inconsistent results. Specific guidelines for antimicrobial susceptibility testing of *Oligella* lack but the incubation could require up to 48 h of incubation. In contrast to *O. urethralis*, which is susceptible to third-generation cephalosporin, *O. ureolytica* is likely resistant to numerous antimicrobials. Genectic determinants of resistance were identified for beta-lactams and aminoglycosides.

**Conclusion:**

*Oligella* is an uncommon pathogen that can be underrecognized. Microbiologists should be prompt to prolong the incubation of agar media plated with urines when the direct examination showed Gram-negative coccobacilli. Carbapenems should probably be given for the empirical treatment.

**Supplementary Information:**

The online version contains supplementary material available at 10.1007/s10096-024-04797-9.

## Introduction

An increasing number of bacterial genera and species were described since bacterial taxonomy is based on a molecular approach. Of them, the genus *Oligella* spp. was described in 1987 [1]. While it was first thought to belong to the urogenital microbiota, the bacterium has been recovered from various samples. Indeed, *Oligella* spp. was sparsely associated with invasive infections in patients with underlying conditions or compromised immune systems [2, 3]. Nevertheless, clinical and microbiological data are sometimes partial in case reports and cases series. Consequently, due to the rarity of *Oligella* spp. infections, there are no standardized treatment guidelines. However, empirical therapy with antibiotics effective against Gram-negative bacteria, such as beta-lactams or fluoroquinolones, is often initiated until susceptibility results are available [4]. Treatment should be individualized based on the site of infection, severity, and susceptibility testing. In order to manage *Oligella* spp. infection an overview of its role in Human infections and its antimicrobial susceptibility is at least required.

We reviewed microbiological characteristics, habitat, place in the Human microbiome, role in infection, and antimicrobial susceptibility of the genus *Oligella*.

## Methods

### Search strategy

Searches in accordance with the Preferred Reporting Items for Systematic Review and Meta-Analysis Statement (PRISMA) guidelines [5] were conducted in PubMed/Medline among studies published from inception to and including October 31, 2023. The following search strategy was used: (*Oligella*) OR (*Moraxella urethralis*) OR (CDC group IVe).

### Inclusion and exclusion criteria

Case reports, case series, cohort studies, and clinical trials on *Oligella* spp. infections were eligible as well as all reports describing *Oligella* site of isolation, characters and antimicrobial susceptibility. Isolates with ambiguous identification (i.e. not reaching the criteria for accurate identification for the method) were excluded. Studies in languages other than English were not eligible.

### Study selection

**Fig. 1:**
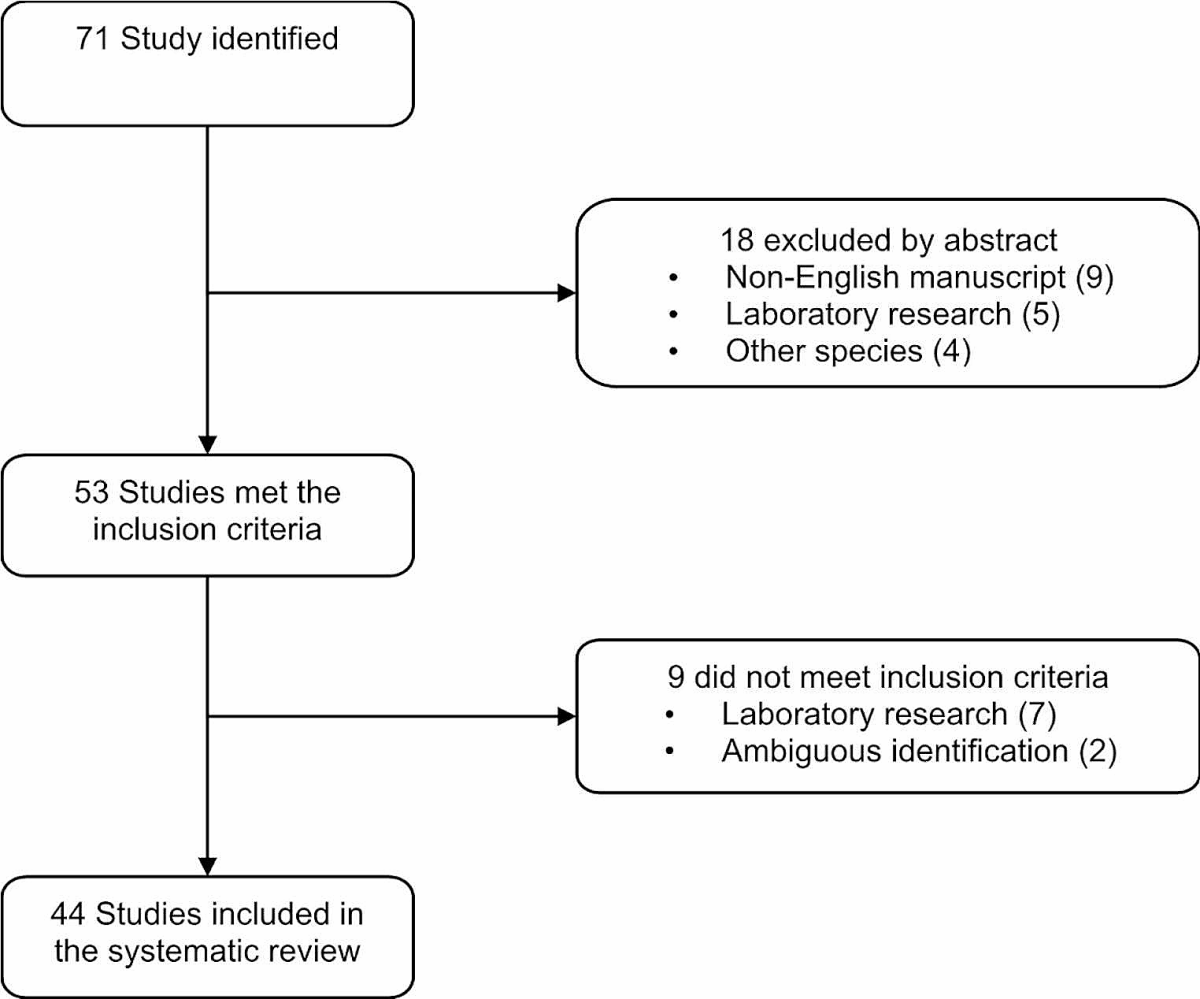
PRISMA flow diagram

The author performed the literature search. Conforming to the PRISMA guidelines, firstly titles or abstracts were screened. Relevant publications were identified, the full text was read and assessment was based on the inclusion and exclusion criteria previously mentioned (Fig. 1). Using the eligibility criteria, EF and AV independently screened all articles and abstracts and reviewed the full text of potentially eligible abstracts.

### Data extraction

The following data were extracted from each eligible article: socio-demographic characteristics, the bacterial species, the type of infection, predisposing conditions, associated micro-organisms, and outcome. Data relative to the site of isolation and the microbiological characters of the isolate (i.e. phenotypic biochemical, antimicrobial susceptibility) were also extracted.

### Risk of Bias

To assess the quality of included studies, the Joanna Briggs Institute (JBI) critical appraisal checklist for case reports was performed [6]. Risk of bias is shown in Supplemental File 1.

## Results

### Taxonomy and microbiological characteristics

The genus *Oligella* belongs to the family Alcaligenaceae. It comprises two species, *Oligella urethralis* (formerly *Moraxella urethralis*) and *Oligella ureolytica* (formerly CDC group IVe) [1]. The closest relatives are *Taylorella equigenitalis* and members of the family Alcaligenaceae. Nevertheless, the taxonomic position of *Oligella* was based on DNA-DNA hybridization or DNA-rRNA hybridization [1]. A more recent approach based on whole genome sequencing would probably precise the taxonomic position of *Oligella* [7].

*Oligella* are aerobic, oxidase-positive, catalase-positive, non-fermentative Gram-negative coccobacilli [4, 8]. *O. urethralis* is non-motile while *O. ureolytica* is motile by means of long peritrichous flagella [1]. Cultural and biochemical characteristics of *O. urethralis* and *O. ureolytica* are listed in Table 1 [1, 9–11]. . Both species form millimetric, non-hemolytic, non-pigmented, colonies on Columbia agar supplemented with 5% horse blood or chocolate agar after 48 h of incubation at 35 °C in aerobic conditions [4]. Almost all strains cultivate on Mac Conkey agar. Some strains of *O. urethralis* are able to grow at 25 and 42 °C, while *O. ureolytica* does not grow at 42 °C [1, 9, 12]. Both species did not ferment sugar and are positive for nitrite reduction reaction, while indol, and gelatinase activities are negative. *O. urethralis* and *O. ureolytica* could be differentiated using the following characters: urease, nitrate reduction, and *p*-Hydroxybenzoate.

**Table 1:** Sample of origin of *Oligella* spp. strains

Origin	Country	Reference
Human		
Blood	France, India, Italy, Turkey, U.S.	[2, 11, 14, 16, 17, 39, 40, 47]
Lymph node	Canada	[3]
Brain stimulator	U.S.	[42]
Cerebrospinal fluid (contaminated)	Sweden	[1]
Joint	U.S.	[37]
ENT (Ear Nose and Throat)	Australia, France, the Philippines, Sweden, United Kingdom, U.S.	[1, 11, 27, 28, 47]
Respiratory tract	France	[4]
Urine	Japan, U.S.	[1, 11, 18]
Genital	Canada, Denmark, U.S.	[1, 11]
Peritoneal dialysis fluid	United Kingdom	[12]
Feces	China	[29]
Wound	Sweden, U.S.	[1, 11]
Animal		
Donkeys (*Equus asinus*)	U.S.	[24]
Camel (*Camelus dromedaries*)	Saudi Arabia	[15]
Cattle	Israel	[25]
Rabbit (*Oryctolagus cuniculus*)	U.S.	[26]
Sprague-Dawley rats	China	[33–35]
Rat	China	[48]
Croiler chickens	Hungary	[49]
Environment		
Permanent makeup tattoo inks	U.S.	[20]
Pistol pump automated injection system	Germany	[21]
Bulk tank milk from dairy herds	U.S.	[22]
Kitchen Waste	China	[50]
Water reservoir	Poland	[23]

### Identification

The performances of laboratory methods of identification were almost never assessed as part of specific studies. Nevertheless, some isolates from case reports were identified using one or more methods. Biochemical methods could provide incomplete or inconsistent identification. The API 32GN was reported once to provide identification to the genus level of an isolate of *O. ureolytica* [2]. Using the Minitek system, *O. urethralis* was likely misidentified, whilst *O. ureolytica* is correctly identified with sometimes the need for additional tests [13]. Yamaguchi et al. reported the precise identification of a clinical isolate of *O. urethralis* was difficult using the MicroScan Walkaway 96 Plus (Beckman Coulter). Conversely, some isolates of both species were identified using the Vitek systems (BioMérieux) with a high probability score [2, 14–17]. Matrix-assisted laser desorption ionization-time of flight (MALDI-TOF) mass spectrometry performances were never assessed for *Oligella* species, but the identification of at least 2 isolates of *O. urethralis* using MALDI-TOF mass spectrometry was confirmed using 16 S rRNA gene sequencing [4, 18].

### Habitat

*Oligella* spp. has been recovered from a wide range of human, animal, and environmental samples worldwide (Table 2). Among a collection of 28 *O. urethralis* received by the American Center for Disease Control (CDC), 16 (57.1%) and 3 (10.8%) were from urine and genital samples respectively suggesting the bacteria is a commensal of the urogenital tract [11]. Furthermore, *Oligella urethralis* was recovered from more than 10% of urine samples of 60 women with urgency urinary incontinence in the absence of clinical infection [19].

**Table 2:** Biochemical and cultural characteristics of *O. urethralis* and *O. ureolytica*

Refences	O. urethralis					O. ureolytica	
	[1]	[1]	[9]	[10]	[11]	[1]	[1]
No. of strains	1 (strain ATCC17960)	14	22	59	22	1 (strain CDC C379)	11
Motility	-*	-				+	+
Cultural characteristic							
Growth Mac-Conkey agar			v (96%)	+	+		
Growth SS agar				-			
Growth Cetrimide agar				-			
Growth at 25 °C			v (50%)	v (93%)			
Growth at 35 °C			+	+			
Growth at 42 °C	+	+	v (59%)	v (88%)		-	v (27.3%)
Growth with 4.5% NaCl	+	v (57.1%)				+	+
Beta-hemolysis			-		-		
Biochemical characteristics							
Oxydase			+	+	+		
Catalase			+	+	v (33%)		
Glucose			-	-	-		
Xylose			-	-			
Lactose			-	-			
Mannitol			-	-			
Sucrose				-			
Maltose				-			
D-Malate	+	v (92.8%)				-	v (36.4%)
Itaconate	+	+				+	+
Isovalerate	+	v (85.7%)				+	v (90.9%)
Isobutyrate	+	v (92.8%)				-	v (54.5%)
*p*-Hydroxybenzoate	-	-				+	+
*m*-Hydroxybenzoate	-	v (7.1%)				-	v (36.4%)
*o*-Hydroxybenzoate	-	-				-	v (9.1%)
Glutarate	+	+				+	v (90.9%)
Mesaconate	-	v (85.7%)				+	v (90.9%)
Phenylacetate	-	-				-	v (45.4%)
Propionate	+	+				+	v (81.8%)
L-Tartrate	-	v (14.2%)				+	v (18.2%)
n-valerate	+	v (92.8%)				+	v (90.9%)
D-α-Alanine	+	v (92.8%)				+	v (90.9%)
L-α-Alanine	+	v (71.4%)				-	v (18.2%)
DL-2-Aminobutyrate	-	-				-	v (9.1%)
DL-4-Aminobutyrate	-	-				-	v (9.1%)
DL-5-Aminovalerate	+	v (92.8%)				-	v (9.1%)
L-Lsoleucine	+	v (42.8%)				-	v (9.1%)
L-Leucine	+	v (57.1%)				-	v (18.2%)
L-Norleucine	+	v (71.4%)				-	v (45.4%)
DL-Norvaline	-	v (21.4%)				-	-
L-Proline	+	+				+	v (72.7%)
Trigonellin	-	-				-	v (9.1%)
L-valine	+	v (21.4%)				-	-
Glycerol				-			
Citrate	-	v (64.3%)		v (92%)		+	+
Nitrate reduction	-	-	-	-	-	+	v (81.8%)
Nitrite reduction	+	v (85.7%)		+	+	+	v (81.8%)
Indol				-	-		
Methyl red				-			
Voges Proskauer				-			
Urease	-	-	-	-	-	+	+
Gelatinase			-	-	-		
Phenylalanine deaminase				+	+		

* +: positive; -: negative; v: inconstant; the rate of isolates displaying the character are in brackets

*Oligella* was also isolated from environmental sources and manufactured products. The U.S. Food and Drug Administration identified microbial contamination in 49% (42 over 85) of unopened tattoo and permanent makeup inks purchased in the U.S [20]. . Of the bacterial contaminant, *Oligella ureolytica* was recovered from one unopened tattoo ink [20]. The pistol pump automated injection system used for the injection of contrast agent/saline solution for magnetic resonance imaging could be at risk of bacterial contamination. In Germany, bacterial contamination was assessed at 3.9% among 205 samples, of which *O. ureolytica* was recovered once [21]. *Oligella urethralis* was also recovered from 1 (0.8%) of 131 bulk tank milk from dairy herds in eastern South Dakota and western Minnesota [22]. *Oligella* spp. was isolated from a water reservoir in Poland [23].

In animals, *O. urethralis* was recovered from the external genitalia of 11.6% of 43 donkeys (*Equus asinus*) [24], ovarian hydrobursitis of a female camel (*Camelus dromedaries*) [15], urinary tract infection in dairy cattle [25], conjunctiva of rabbit (*Oryctolagus cuniculus*) [26]. *O. ureolytica* has never been isolated from animals to date.

### Microbiome

#### Ear microbiota

Using a genomic approach based on 16 S rRNA gene sequencing, Taylor et al. assessed the microbiota of Australian aborigines with otitis media. Participants were 19 children having a median age of 3.2 years (extremes 3 months − 7 years) [27]. *Oligella* spp. was found to be significantly more abundant in children having ear disease with perforation, detected in 16% of all ear swabs. Relative abundance reached up to 60% in a 2-years old child with acute otitis media with perforation [27]. Furthermore, Using a 16 S rRNA gene sequencing, *Oligella* was also identified in the outer ear and middle ear swabs of indigenous Filipino children [28]. Sixteen children were included in the study of which 11 carry the *A2ML1* variants gene that is associated with otitis media susceptibility. *Oligella* spp. was detected in the outer and/or the middle ear of thirteen (81.3%) children [28]. In this population, *Oligella* spp. relative abundance was higher than that of *Corynebacterium* spp [28].

#### Digestive microbiota

Relatedness of gut microbiota and subclinical carotid arterial atherosclerosis was assessed in a study including 569 asymptomatic elderly in rural China [29]. Subclinical carotid arterial atherosclerosis was significantly associated with gut microbiota and lifestyles [29]. Indeed, lifestyle and diet are associated with atherosclerosis and cardiovascular diseases [30–32]. In Zhu et al. study, fecal metagenomic analysis revealed a down-regulating abundance of 3 bacterial genera, *i.e. Oligella*, *Alistepes*, and *Prevotella*, that was correlated to taking more fresh aquatic food, vegetables, fruits, and doing more exercise [29]. The gut microbes explained 16.5% of the mediation effect of lifestyles on the pathogenesis of carotid atherosclerosis [29].

Using a model of high-fat diet-induced obesity of rats, banana-resistant starch was shown to reduce the abundance of 3 bacterial genera, *Oligella*, *Turicibacter*, and *Romboutsia*, and increased that of *Bacteroides*, *Ruminococcaceae*, and *Lachnospiraceae* [33]. Whilst, supplementation of young rat food with compound polysaccharides significantly increased the abundance of 4 bacterial genera (*Bifidobacterium, Lactobacillus, Allobaculum*, and *Oligella*) and was associated with the development of the metabolic activity of intestinal microbiota [34]. In another study, the impact of fecal microbiota on nephropathy induced by hyperuricemia was assessed using an experimental model of rats based on the administration of a large amount of urate precursors [35]. The gut microbiota was significantly changed compared with the control group: *Flavobacterium*, *Myroides*, *Corynebacterium, Alcaligenaceae*, and *Oligella* increased significantly while *Blautia* and *Roseburia* were greatly reduced.

#### Urogenital microbiota

Urine is a low microbial biomass environment and its analysis requires specific technical considerations [36]. Thus, sensitive extraction methods are required to obtain good quality DNA and detect low-abundant bacteria [36]. The detection of *Oligella* spp. might in urine sample using a molecular approach might therefore depends on the method used.

While *Oligella* was first thought to be commensal of the urogenital tract, a single study reports their place in urinary microbiota. Pearce et al. compare the female urinary microbiome in 60 women with urgency urinary incontinence in the absence of clinical infection in comparison to 58 women without urgency urinary incontinence [19]. They found *Oligella urethralis* was only detected in the urine of women with urgency urinary incontinence. Overall, *Oligella* was cultured from more than 10% of the group with urgency urinary incontinence [19].

### Infections in human

We retrieved 14 infections involving *Oligella* spp (Table 3). The median age was 51 years old [extremes 0–88] and the male/female ratio was 1.8. *O. urethralis* and *O. ureolytica* were involved in 6 and 7 cases respectively, the remaining isolate was identified to the genus level. All-but-one infection involved *Oligella* spp. alone. Almost all patients had predisposing conditions. The types of infections were: 1 pulmonary abscess (*O. urethralis*) [4], 1 Urosepsis (*O. urethralis*) [18], 2 chronic ambulatory peritoneal dialysis peritonitis (*O. urethralis*) [12], 1 Knee septic arthritis (*O. urethralis*) [37], 1 lymph node infection (*O. ureolytica*) [3], and 7 primary bacteremia (1 and 6 due to *O. urethralis* and *O. ureolytica* respectively) [2, 14, 16, 17, 38–40]. Invasive infections could suggest *Oligella* spp. display some virulence factors that could contribute to dissemination in patients with comorbidities. In all cases of primary bacteremia, urine samples were negative in culture [2, 14, 16, 17, 38–40]. Nevertheless, *Oligella* spp. are slow-growing bacteria that could require at least two days of incubation [4], whilst urine samples handled in clinical laboratories are usually incubated for 24 h. Consequently, an urosepsis could not be excluded. Furthermore, rarely encountered micro-organisms such *Oligella* spp. are usually not reported in studies assessing the prevalence of micro-organisms in UTI [41], and non-invasive infections are likely not reported in the literature. Therefore, the prevalence of *Oligella* as a urinary tract pathogen remains unclear.

**Table 3:** Reported infections involving *O. urethralis* and *O. ureolytica*

No	Age	Sex	Species	Infection	Predisposing conditions	Other microorganisms	Medical history	Outcome	Ref.
1	51	M	*O. urethralis*	Pulmonary abscess	• Chronic alcohol abuse • Cigarette smoking • Stage II chronic obstructive pulmonary disease • Locally advanced but stable non-small-cell lung cancer	No	• On admission: respiratory distress, altered mental status, hypercapnia with profuse sweating, and tachycardia, which rapidly worsened (Glasgow coma scale 14 to 3). Chest computed tomography (CT) showed pulmonary abscess of the entire lower-left lobe. • IV cefotaxime and rovamycin started. • Day 4: Transfert to intensive care unit. • Day 5: death from massive hemoptysis • Protected bronchial sample showed Gram-negative coccobacilli on Gram-stain Culture grew *O. urethralis* identified using MALDI-TOF mass spectrometry and 16 S rRNA gene sequencing.	Death	[4]
2	69	M	*O. urethralis*	Chronic ambulatory peritoneal dialysis peritonitis	• Non-insulin dependent diabetes mellitus with a 1-year history of end-stage renal failure • Chronic Ambulatory Peritoneal Dialysis Peritonitis	No	• Admitted with abdominal pain and confusion for 4 days. • Peritoneal dialysate on admission was cloudy and showed 1230 white blood cells/mm 90% of which were neutrophils. • Empirically started on IV flucloxacillin and ciprofloxacin switched for ampicillin and gentamicin 2 days later. • Dialysate direct culture was negative, but dialysate inoculated into blood culture bottles for enrichment yielded *O. urethralis.* • Relief of symptoms with the removal of the Tenchkoff catheter	n.a.	[12]
3	29	M	*O. urethralis*	Chronic ambulatory peritoneal dialysis peritonitis	• End-stage renal failure of uncertain aetiology • Two failed cadaveric renal transplants, both removed • Tenchkoff catheter inserted 3 years previously but Chronic ambulatory peritoneal dialysis abandoned for haemodialysis after 2 years because of poor drainage	No	• Admitted for 12 h history of abdominal pain and fever. • Tenchkoff catheter split at the bung site • Started empirically on IV vancomycin and oral ciprofioxacin and the catheter re- moved. His symptoms settled 2 days after admission. • Direct culture grew Corynebacterium spp. Fluid enrichment culture in blood culture bottles grew Corynebacterium spp. and O. urethralis.	n.a.	[12]
4	90	F	*O. urethralis*	Urosepsis secondary to emphysematous pyelonephritis	• Diabetes mellitus • Levofloxacin administration in the past two-month, reason not available.	No	• Admitted for single-day fever and impaired consciousness. Diagnosis of septic shock with unknown focus. Empirical Meropenem IV administered. • Abdominal CT showed hydronephrosis and pneumatosis with renal stones in the left kidney • Diagnosis of urosepsis secondary to emphysematous pyelonephritis. Ureteral stents inserted to both urinary ducts. • Day 4: Blood culture positive for Gram negative rods identified as *O. urethralis* using MALDI-TOF mass spectrometry and 16 S rRNA gene sequencing. Urines from stent also grew *O. urethralis*.	Recovery	[18]
5	83	M	*O. urethralis*	Knee septic arthritis	• Rectum adenocarcinoma,	No	• Admitted for warm and tender right knee greater in circumference • Exploration - Radiographs of the right knee showed signs of chondrocalcinosis. - knee joint fluid grew *O. urethralis* - Blood culture and urines negatives • Treated with amoxicillin 2 g/d for 3 weeks. Fever defervesced in 24 h.	Recovery	[37]
6	75	M	*O. urethralis*	Bacteremia	• Metastatic colorectal carcinoma, • Renal failure with bilateral hydrone- phrosis • Gastrointestinal obstruction secondary to local spread for which bilateral nephrostomies and a colostomy	No	• Admitted for fever, nausea, and vomiting, anuric since a day • Obstruction of the right nephrostomy tube. Change of the left nephrostomy tube • Started on sulbactam/ ampicillin • Blood culture and urines grew *O. urethralis*.	n.a.	[38]
7	66	M	*O. ureolytica*	Bacteremia	• Aortic valve replacement for severe aortic valve stenosis • Arterial hypertension, • non-critical carotidal atherosclerosis, • dyslipidaemia, • fatty liver, • smoking and alcohol abuse • chronic gastritis • groin hernia surgical intervention L4-L5 and L5-S1 herniated discs, scoliosis • asymptomatic prostatic hypertrophy and external urethral meatus substenosis (lichen pla nus)	No	• Admitted for fever, dizziness, weight loss of 3 kg on the last month, and a singular episode of biliary vomiting and diarrhoeic stools many days before. • Empirically started on vancomycin (2 g/d IV), gentamicin (3 mg/kg/d IV), and rifampin (600 mg/d oral) • Blood culture positive for *O. ureolytica* identified using Vitek 2. • Antibiotics switched for piperacillin/tazobactam (14 g/d IV) • Exploration: - Urine sample negative - Abdominal ultrasound and computed tomography scan, and colonoscopy: bilateral simple kidney’s cysts and few intestinal polyps with no other abnormalities - Trans-thoracic echocardiography (T.T.E.): no signs of infective endocarditis	Recovery	[2]
8	0	F	*O. ureolytica*	Bacteremia	• Maternal medical history was unremarkable • Labor and delivery unremarkable	No	• Admitted in ICU at day one of life for sepsis • Empirically started on ampicillin (100 mg/kg/day) and netilmicin (5 mg/kg/day) • Blood culture positive for *O. ureolytica* using Vitek2 Urine sample negative • Antimicrobials switched for netilmicin (5 mg/kg/day) 10 days	Recovery	[14]
9	30	M	*O. ureolytica*	Bacteremia	• Right lung adenocarcinoma with brain metastasis • Tobacco use	No	• Admitted for abdominal distension, decreased urine output, inability to pass stool and flatus and headache for past five days. • Empirically started on ciprofloxacin IV ceftriaxone IV, and metronidazole IV. • Day 4: the patient did not respond; antimicrobials were switched to amoxicillin clavulanate IV and meropenem • Blood culture positive for *O. ureolytica* using Vitek2 • Urine sample negatives.	Recovery	[16]
10	66	F	*O. ureolytica*	Bacteremia	• Fallen, stay 4 days laying in her own urine and feces. • Malnutrition • Tobacco use	No	• Femur fracture, and a right buttock stage III decubitus ulcer. • Microbiological exploration: - Blood culture grew *Oligella ureolytica* identified with Vitek2 compact - Wound cultures *Proteus mirabilis* and *Enterococcus* spp. - Urine culture grew > 100,000 CFU *Escherichia coli*. • Empirically started on vancomycin (1 g/d), aztreonam (6 g/d), and metronidazole (1,5 g/d) • Empirically continue aztreonam for endocarditis after her discharge as the patient’s refusal of a transesophageal echocardiogram and the presence of the uncommon bacterium.	Recovery	[17]
11	40	M	*O. ureolytica*	Bacteremia	• AIDS (CD4 lymphocyte count of 19 × 10 6 /L) chronic diarrhea due to Cryptosporidium species, Kaposi’s sarcoma, thrush, significant wasting	No	• 3 days before admission: started with ciprofloxacin • Admitted for weakness and fever of several days’ duration • Explorations - Ultrasonogram of the liver and gallbladder unremarkable. - Urine culture negative. • Started on vancomycin and ceftazidime. • Blood culture grew *O. ureolytica*. • Antimicrobials switched to tobramycin and ciprofloxacin. His condition improved gradually • 7 days later, his conditions worsened, • Sacral decubitus ulcers had not healed and that they were surrounded by erythema, • Blood culture grew *Candida krusei* Bacteroides ureolyticus and Bacteroides thetaiotaomicron. • Death	Death from other cause	[39]
12	49	F	*O. ureolytica*	Lymph node infection	• Chronic lympho cytic leukemia	No	• Presented with enlarged right posterior cervical lymph node measuring 2 × 1.5 × 1 cm, no fever • Fluid aspirated from the lymph node grew *O. ureolytica* • Antimicrobial with ciprofloxacin for 7 days followed by trimethoprim-sulfametoxazole for 2 weeks, minimal response. • Lmph node increased in size, became more painful and began draining clear fluid. However, culture was negative • Started on cephalexin and chemotherapy	Recovery	[3]
13	24	M	*Oligella spp.*	Brain stimulator infection	• Tourette syndrome diagnosed at age of 5 years • Depression, attention deficit hyperactive disorder, and obsessive-compulsive disorder • First step of deep brain stimulator for uncontrolled tics of the bilateral upper extremities and head refractory to maximal medical management.	• *Corynebacterium* spp. • Coagulase-negative *Staphylococcus*	• First step of deep brain stimulator for uncontrolled tics of the bilateral upper extremities and head refractory to maximal medical management. • During second step (connection of leads to a pulse generator in the chest), 5 weeks later, observation of gelatinous material surrounding the connectors. Explantation of the infected material. The stain showed Gram-positive cocci. • Antimicrobials started vancomycin (1500 mg/d intravenous (IV), cefepime (4 g/d), metronidazole (1.5 g/d), narrowed to IV vancomycin and IV cefepime for 6 weeks • Patient reported he did not been apply surgical wound dressings since his initial DBS surgery.	Recovery	[42]
14	87	F	*O. ureolytica*	Bacteremia	• Vesical neoplasia	No	• Admitted for progressive deterioration of general condition, severe malnutrition, and a septic syndrome of urinary origin. Started on ceftriaxone IV and oxacillin IV • Antimicrobials switched for amoxicillin-clavulanate for suspicion of an infection of the left lung • Day 1: blood culture grew O. Ureolytica • Day 4: antimicrobials switched for cefepime and metronidazole • Day 7: blood culture negative • Day 11: antimicrobial stopped • Day 12: reintroduction of antimicrobials Recused for urinary diversion, due to general condition • Day 17: death	Death	[40]

The outcome was available for 11 patients, 8 recovered with antimicrobials [2, 3, 14, 16–18, 37, 42], 2 dead from *Oligella* infection [4, 40], and the remaining one dead from another infectious cause [39]. Of note, *Oligella ureolytica* had been recovered from a single blood culture vials sampled in a 18-months old children with a diagnosis of pneumonia [43]. He recovered despite being administered with inactive antimicrobials, which makes the authors assume the isolate was a contaminant [43].

### Antimicrobial resistance

Antimicrobial susceptibility testing and interpretation were performed using a wide range of methods and guidelines (Table 4). As *Oligella* spp. are slow-growing organisms, incubation of antimicrobials susceptibility testing was reported to require 48 h of incubation [43].

**Table 4:** Antimicrobial susceptibility of *O. urethralis* and *O. ureolytica*

References	O. urethralis							O. ureolytica						
	[44]	[44]	[4]	[18]	[12]	[38]	[37]	[2]	[14]	[16]	[3]	[39]	[43]	[40]
Method	Agar dilution	Agar dilution	Broth microdilution and Disk diffusion	n.a.***	Disk diffusion, MIC determination n.a.	n.a.	Disk diffusion	Disk diffusion	n.a.	Disk diffusion	Disk diffusion	n.a.	Broth microdilution	Disk diffusion
Interpretation	CLSI	CLSI	EUCAST	n.a.	n.a.	n.a.	n.a.	CLSI	CLSI	CLSI	CLSI	n.a.	n.a.	CA-SFM/EUCAST
No. of strains	3*	1**	1	1	2	1	1	1	1	1	1	1	1	1
Penicillin			0.125 mg/L		≤ 0.125 mg/L								> 8 mg/L	
Ampicillin				≤ 0.5 mg/L	S	R	S	R	I	R	R		> 32 mg/L	
Ampicillin sulbactam													16 mg/L	
Amoxicillin	0.5 mg/L	512 mg/L	0.5 mg/L										> 8 mg/L	I
Amoxicillin clavulanate	0.5 mg/L	256 mg/L					S	S	S					I
Ticarcillin	0.5 mg/L	256 mg/L												R
Ticarcillin clavulanate	0.5 mg/L	16 mg/L										R		R
Piperacillin	0.25 mg/L	16 mg/L			S	R				R	R	R	> 512 mg/L	
Piperacillin tazobactam	0.25 mg/L	16 mg/L						S		R			256 mg/L	R
Cephalosporins not specified							S							
Cephalothin	0.5 mg/L	512 mg/L												
Cefazolin											R			
Cefoxitin	0.25 mg/L	16 mg/L												
Moxalactam	0.12 mg/L	0.12 mg/L												
Cefuroxime	0.25 mg/L	128 mg/L			S				S					
Cefpodoxime													> 1 mg/L	
Ceftriaxone				≤ 0.5 mg/L				R	S	R	R			R
Ceftazidime	0.12 mg/L	0.5 mg/L			S				S	R	S	R	32 mg/L	R
Cefotaxime	0.12 mg/L	0.12 mg/L	0.19 mg/L		0.5 mg/L								> 32 mg/L	
Cefepime	0.12 mg/L	0.06 mg/L												S
Cefpirome	0.12 mg/L	0.06 mg/L												
Aztreonam	0.12 mg/L	0.06 mg/L			S							R		R
Imipenem	0.12 mg/L	0.06 mg/L	S		S	S		S	S	S		S	8 mg/L	S
Meropenem				≤ 0.25 mg/L					S	S			> 16 mg/L	S
Pefloxacin							S							
Ciprofloxacin			32 mg/L		> 8 mg/L	R			S	R	S	S	> 2 mg/L	
Ofloxacin					> 8 mg/L								4 mg/L	
Levofloxacin				> 4 mg/L				S					2 mg/L	
Garenoxacin				> 4 mg/L										
Tosufloxacin				> 2 mg/L										
Trimethoprim-sulfamethoxazole			S	≤ 10 mg/L	1 mg/L	S	S		R	R	S	S	> 4 mg/L	S
Aminoglycosides not specified						S	S							
Tobramycin										R		S	≤ 5 mg/L	S
Amikacin										R	S		2 mg/L	S
Gentamycin			0.25 mg/L		S				S			S	≤ 5 mg/L	S
Erythromycin							S						8 mg/L	
Clindamycin							R						> 1 mg/L	
Clarithromycin				≤ 8 mg/L										
Minocycline				≤ 1 mg/L										
Tetracycline					< 1 mg/L								0.25 mg/L	
Rifampicin													4 mg/L	S
Chloramphenicol					< 4 mg/L								8 mg/L	

* *Reference strains* CIP102456, CIP116103, and CIP8133

** *Clinical strain COH-1*

*** n.a. not available

*O. urethralis* is intrinsically susceptible to penicillins, cephalosporins, and carbapenems. Nevertheless, resistant isolates were described. Strain COH-1 was described to harbor two chromosomal genes encoding β-lactamases: *bla*_ABA−1_, an AmpC cephalosporinase gene from *Acinetobacter baumannii*, and *bla*_CARB−8_ [44]. Since *O. urethralis* reference strains CIP102456, CIP116103, and CIP8133 did not harbor these genes confirm chromosomal integration in strain COH-1 [44]. *bla*_ABA−1_ was subsequently renamed *bla*_ADC−2_, the ADC class of β-lactamases was far described for *Acinetobacter baumannii* and *Acinetobacter* spp [45]. . Most strains of *O. urethralis* were rested resistant to fluoroquinolones while aminoglycosides and trimethoprim-sulfamethoxazole combination appeared active in vitro.

In contrast to *O. urethralis*, all strains of *O. ureolytica* display decreased susceptibility to ampicillin or amoxicillin suggesting *O. ureolytica* harbor a chromosomal encoding penicillinase gene. Some isolates were found susceptible to penicillin – penicillinase inhibitor combination. Third-generation cephalosporins were active against a single strain of *O. ureolytica* [14], while all-but-one strains were susceptible to carbapenems in vitro. The single carbapenem-resistant strain was resistant to all β-lactams using the broth microdilution method with prolonged incubation time (48 h) [43]. Imipenem and meropenem MIC were 8 mg/L and > 16 mg/L respectively. The mechanisms of resistance to β-lactams including carbapenems remain to be assessed for *O. ureolytica*. Fluoroquinolones, aminoglycosides, and trimethoprim-sulfamethoxazole showed inconsistent activity in vitro against *O. ureolytica*. Resistance to aminoglycosides was mediated by an aminoglycosides acetyl-transferase gene (*acc(6’)-Ib*) in an isolate of *O. ureolytica* from the urinary tract of a children in Iraq [46]. 

## Conclusion

The genus *Oligella* comprises two species that were recovered from various samples worldwide. In humans, they are mainly found as part of the microbiome of individuals with predisposing conditions. But, *Oligella* were also recovered from environmental source and *O. uretrhalis* was isolated from animals. *Oligella* were mainly associated with invasive infections in patients with predisposing conditions. But their prevalence, including in urinary infections, remains to determine. Indeed, as they are uncommon, they are likely not reported in epidemiological studies. Their identification in the clinical laboratory is easy using MALDI-TOF mass spectrometry, but they could require up to 48 h to grow in vitro. Consequently, microbiologists should be prompt to prolong the incubation of agar media plated with urines when the direct examination showed Gram-negative coccobacilli. *O. urethralis* could acquire genes encoding antimicrobial resistance. *O. urethralis* is susceptible to third-generation cephalosporin. Conversely, *O. ureolytica* is likely highly resistant to antimicrobials. Carbapenems should probably be given for the empirical treatment.

However, since the bibliographic search mainly retrieved case reports and case cohorts, more studies with robust methodology including should be conducted to assess the prevalence of *Oligella* as a uropathogen. The factors associated with the risk of infection, and the mechanisms of resistance to antimicrobials remain to be clarified.

### Electronic supplementary material

Below is the link to the electronic supplementary material.

Supplementary Material 1

## Data Availability

Not applicable.
