# Supplementary material for: Oligella spp.: A systematic review on an uncommon urinary pathogen
Source: Eur J Clin Microbiol Infect Dis. 2024 Apr 26;43(6):1037–50. doi: 10.1007/s10096-024-04797-9 (PMC11178559; doi:10.1007/s10096-024-04797-9)
Supplement: Supplementary file 1 — Supplementary Material 1 [file 10096_2024_4797_MOESM1_ESM.docx]

Supplementary Table 1. The Joanna Briggs Institute (JBI) Critical Appraisal Checklist for Case Reports

| Reference | 1. Were patient’s demographic characteristics clearly described? | 2. Was the patient’s history clearly described and presented as a timeline? | 3. Was the current clinical condition of the patient on presentation clearly described? | 4. Were diagnostic tests or assessment methods and the results clearly described? | 5. Was the intervention(s) or treatment procedure(s) clearly described? | 6. Was the post-intervention clinical condition clearly described? | 7. Were adverse events (harms) or unanticipated events identified and described? | 8. Does the case report provide takeaway lessons? |
| --- | --- | --- | --- | --- | --- | --- | --- | --- |
| 2 | Yes | Yes | Yes | Yes | Yes | Yes | N/A | Yes |
| 3 | Yes | Yes | Yes | No | Yes | Yes | N/A | Yes |
| 4 | Yes | Yes | Yes | Yes | Yes | Yes | N/A | Yes |
| 12 | Yes | Yes | Yes | Yes | Yes | Yes | N/A | Yes |
| 14 | Yes | Yes | Yes | Yes | Yes | Yes | N/A | Yes |
| 16 | Yes | Yes | Yes | Yes | Yes | Yes | N/A | Yes |
| 17 | Yes | Yes | Yes | Yes | Yes | Yes | N/A | Yes |
| 18 | Yes | Yes | Yes | Yes | Yes | Yes | N/A | Yes |
| 37 | Yes | Yes | Yes | Yes | Yes | Yes | N/A | Yes |
| 38 | Yes | Yes | Yes | No | Yes | No | N/A | Yes |
| 39 | Yes | Yes | Yes | No | Yes | Yes | N/A | Yes |
| 40 | Yes | Yes | Yes | Yes | Yes | Yes | N/A | Yes |
| 42 | Yes | Yes | Yes | No | Yes | Yes | N/A | Yes |
